# Analysis of Differences in Characteristics of High-Risk Endemic Areas for Contracting Japanese Spotted Fever, Tsutsugamushi Disease, and Severe Fever With Thrombocytopenia Syndrome

**DOI:** 10.1093/ofid/ofae025

**Published:** 2024-01-16

**Authors:** Takahisa Ogawa, Shinya Tsuzuki, Hiroyuki Ohbe, Hiroki Matsui, Kiyohide Fushimi, Hideo Yasunaga, Satoshi Kutsuna

**Affiliations:** Department of Orthopedic, Saku General Hospital Saku Medical Center, Nagano, Japan; Department of Health Policy and Informatics, Tokyo Medical and Dental University, Tokyo, Japan; Disease Control and Prevention Center, National Center for Global Health and Medicine, Tokyo, Japan; Faculty of Medicine and Health Sciences, University of Antwerp, Antwerp, Belgium; Department of Clinical Epidemiology and Health Economics, School of Public Health, The University of Tokyo, Tokyo, Japan; Department of Clinical Epidemiology and Health Economics, School of Public Health, The University of Tokyo, Tokyo, Japan; Department of Health Policy and Informatics, Tokyo Medical and Dental University, Tokyo, Japan; Department of Clinical Epidemiology and Health Economics, School of Public Health, The University of Tokyo, Tokyo, Japan; Department of Infection Control and Prevention, Graduate School of Medicine, Faculty of Medicine, Osaka University, Osaka, Japan

**Keywords:** ecologic study, mixed-effect model, tick-borne disease

## Abstract

**Background:**

Tick-borne infections, including tsutsugamushi disease, Japanese spotted fever, and severe fever with thrombocytopenia syndrome (SFTS), are prevalent in East Asia with varying geographic distribution and seasonality. This study aimed to investigate the differences in the characteristics among endemic areas for contracting each infection.

**Methods:**

We conducted an ecologic study in Japan, using data from a nationwide inpatient database and publicly available geospatial data. We identified 4493 patients who were hospitalized for tick-borne infections between July 2010 and March 2021. Mixed-effects modified Poisson regression analysis was used to identify factors associated with a higher risk of contracting each tick-borne disease (Tsutsugamushi, Japanese spotted fever, and SFTS).

**Results:**

Mixed-effects modified Poisson regression analysis revealed that environmental factors, such as temperature, sunlight duration, elevation, precipitation, and vegetation, were associated with the risk of contracting these diseases. Tsutsugamushi disease was positively associated with higher temperatures, farms, and forests, whereas Japanese spotted fever and SFTS were positively associated with higher solar radiation and forests.

**Conclusions:**

Our findings from this ecologic study indicate that different environmental factors play a significant role in the risk of transmission of tick-borne infections. Understanding the differences can aid in identifying high-risk areas and developing public health strategies for infection prevention. Further research is needed to address causal relationships.

## Teaser

This nationwide study of 4493 patients hospitalized for tick-borne infections explored environmental factors associated with the diseases. Tsutsugamushi disease was positively associated with higher temperatures, farms, and forests, whereas Japanese spotted fever and severe fever with thrombocytopenia syndrome were positively associated with higher solar radiation and forests.

Tick-borne infections are endemic worldwide, and many emerging infectious diseases have been identified in recent years. Three tick-borne infectious diseases are prevalent simultaneously in East Asia: tsutsugamushi disease, Japanese spotted fever, and severe fever with thrombocytopenia syndrome (SFTS) [1–4]. The epidemiology of tick-borne infectious diseases in Japan varies from disease to disease. In Japan, tsutsugamushi disease has been reported throughout the country except in Hokkaido, while cases of Japanese spotted fever and SFTS have mainly been reported in western Japan. This may be occurring because the ticks carrying Japanese spotted fever and SFTS are unevenly distributed in western Japan [5, 6].

However, these infectious disease hotspots do not always coincide. Additionally, their geographic distribution and seasonality are not always consistent [7]. One reason for this may be the different vectors transmitting these infections. The vectors of *Rickettsia japonica*, the pathogen of Japanese spotted fever, are reported as *Haemaphysalis flava*, *Haemaphysalis longicornis*, and *Ixodes ovatus* [8–10]. *Orientia tsutsugamushi*, the causative microorganism of tsutsugamushi disease, is transmitted by chiggers [11]. SFTS virus is transmitted by ticks, such as *Haemaphysalis longicornis* and *Amblyomma testudinarium*.

In addition to these vector differences, the distribution of host mammals in the region and other factors are likely to result in different endemic areas for each infection. We noted these differences and hypothesized that there may be regional characteristics enhancing the susceptibility of each tick vector to tick-borne infection, such as elevation and average temperature. The purpose of our study was to investigate the characteristics of the endemic areas of each tick-borne infection and analyze the differences among endemic areas of transmission of these infections.

## METHODS

### Settings and Data Source

This ecologic study used data from a nationwide inpatient database and publicly available geospatial data from Japan.

#### Individual Patient Information

To obtain the number of cases of tick-borne infections in a given area, we used the Japanese Diagnosis Procedure Combination (DPC) inpatient database, which contains discharge abstracts and administrative claims data of >1200 acute care hospitals that voluntarily contribute to the database in Japan [12]. The coverage rate of inpatients in this database over all acute care inpatients in Japan exceeded 50%. The database covers approximately 90% of all tertiary care emergency hospitals and 80% of the institutions certified by the Japanese Association for Infectious Diseases for training board specialists. A previous validation study of this database showed high specificity and moderate sensitivity for diagnoses and high specificity and sensitivity for procedures [13].

Using the Japanese DPC inpatient database from July 2010 to March 2021, we identified all patients hospitalized for tick-borne infections based on *ICD-10* codes A68, A69, A75, A77, A79, A84, A93, B60, and B88 (Supplementary Table 1). We excluded patients with a suspected diagnosis of tick-borne infection. We also identified patients hospitalized for Japanese spotted fever (*ICD-10* code A778), tsutsugamushi disease (code A753), and SFTS (code A938). We obtained information about the patients’ residential areas using aggregated city code–level data based on the zip codes of the patients’ residential areas to calculate the number of cases in an area during the observation period. In calculating the number of cases, we assumed that nearly all patients who developed a tick-borne infectious disease would have been admitted to tertiary care emergency hospitals or institutions certified by the Japanese Association for Infectious Diseases for training board specialists.

#### Weather and Geospatial Information

To obtain geospatial information, we used the Geographic Information System (GIS) information provided in Japan [14]. Weather information, such as temperature, sunlight, and precipitation, was obtained from the Japan Meteorological Agency [15].

Altitude information was averaged for each city code area, and types of landscape were used as the most frequent values for the area (Supplementary Table 2). For example, in each city code area, the landscape area was the most frequent, followed by mountains and agricultural areas; therefore, we used landscape as the most frequent value of the area. To consider the differences in the number of potential patients at risk of infection in each city code area, we used the Japanese census data for the area.

#### Spatial Analysis

First, individual patients’ data were obtained from the DPC database, which includes patient zip code information. We then obtained geospatial information and aggregated the zip code–level data into a 5-digit Japanese local government city level code (developed by the Ministry of Internal Affairs and Communications). We did so because several geospatial parameters were available at only the code level. Finally, the patients’ individual data and geospatial information were merged by city code [16].

### Statistical Analysis

Mixed-effects modified Poisson regression analysis was used to identify factors associated with a higher risk of contracting each tick-borne disease (Tsutsugamushi, Japanese spotted fever, and SFTS). Poisson regression is considered appropriate for analyzing rare events. When it is applied to binomial data, the error in the estimated relative risk is overestimated [17]. This problem may be rectified by using a robust error variance procedure known as sandwich estimation or modified Poisson regression [18].

Each Poisson regression model included the number of cases in each subdistrict, defined by the GIS data as the dependent variable. For the independent variables, we included monthly mean temperature, mean solar radiation, monthly total precipitation, mean elevation, and vegetation (forest, farm, or others) in each subdistrict as fixed effects and only population in each district as the random effect. Climate and elevation data were classified into 3 tertile categories. We defined temperature, solar radiation, precipitation, and elevation by 3 quartiles (low, middle, and high) and used middle values as a reference in the model. Each variable was divided into the following ranges: temperature (Celsius; low, −1.1 to 11.6; middle, 11.7–15.0; high, 15.1–23.9), solar radiation (MJ/m^2^; low, 11.0–12.8; middle, 12.9–13.4; high, 13.5–15.9), precipitation (mm; low, 675–1405; middle, 1406–1851; high, 1852–3946), and elevation (m; low, 1.4–9.7; middle, 9.8–29.7; high, 29.8–113.9; Supplementary Table 3). All variables defined by the GIS data were classified into 20 population scale clusters.

Two-sided *P* values <.05 were considered statistically significant. All analyses were conducted with R version 4.1.3 (R Core Team), Stata/SE version 17.0 (Stata Corp), and QGIS version 3.22 (Quantum GIS Development Team).

### Ethical Approval

This study was approved by the Institutional Review Board of the University of Tokyo (approval 3501-3; 25 December 2017). No identifying information of individual patients, hospitals, or physicians was obtained, and the requirement for obtaining informed consent was waived because of the anonymized nature of the data.

## RESULTS

**Figure 1.**
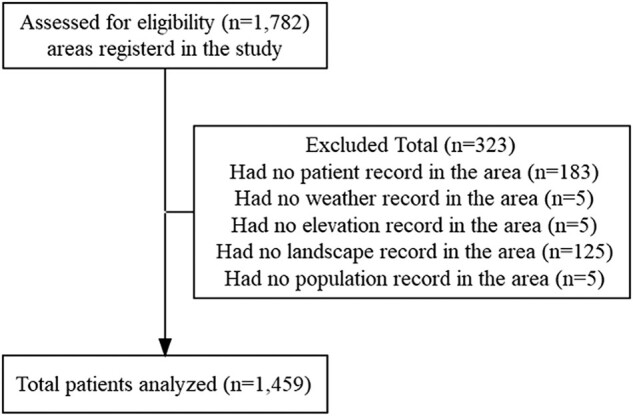
Flowchart of patient selection.

We identified 4493 patients (mean ± SD age, 64.3 ± 19.5 years; male, 53.9%) who were hospitalized for tick-borne infections during the study period. The number of patients hospitalized for Japanese spotted fever, tsutsugamushi disease, and SFTS was 1386 (30.9%), 1576 (35.1%), and 451 (10.0%), respectively. The overall in-hospital mortality rate was 2.7% (121/4372). The in-hospital mortality rates of patients hospitalized for Japanese spotted fever, tsutsugamushi disease, and SFTS were 2.0% (27/1386), 0.5% (8/1586), and 18.4% (83/368). Figure 1 illustrates a flowchart of patient selection.

**Figure 2.**
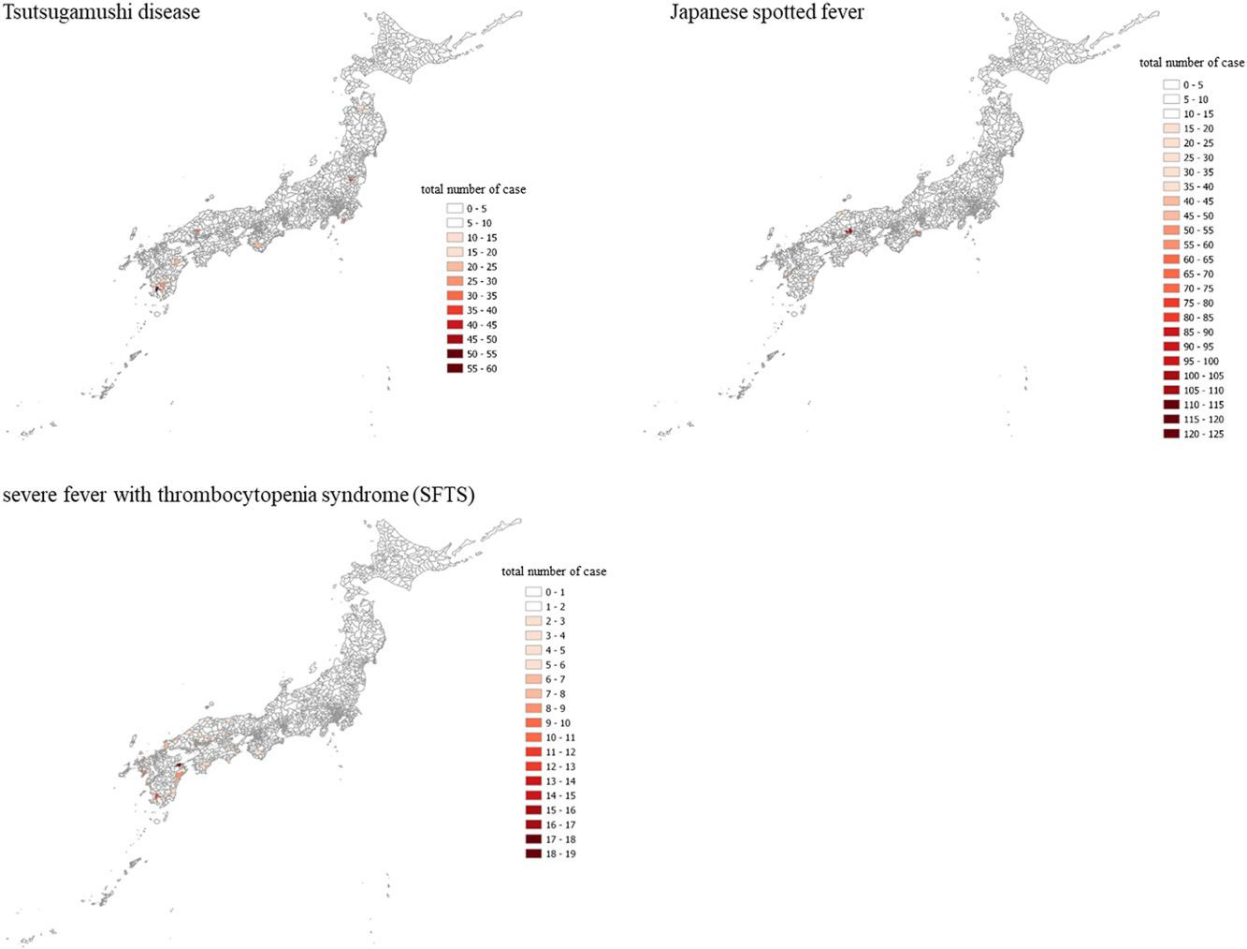
Distribution of total number of tick-borne disease cases across Japan over the observational period: Tsutsugamushi, Japanese spotted fever, and severe fever with thrombocytopenia syndrome.

The distribution of all patients is shown in Figure 2. The average temperature, sunlight, and precipitation for each city code area are shown in Supplementary Figure 1. The elevation, vegetation, and population data are shown in Supplementary Figure 2.

Tables 1, 2, and 3 show the association of weather and geospatial parameters with the incidence of hospitalization for tick-borne diseases. The incidence of hospitalization for Tsutsugamushi disease was positively associated with higher temperatures, farms, and forests and negatively with shorter solar radiation, lower elevation, and lower precipitation. The incidence of hospitalization for Japanese spotted fever was positively associated with longer solar radiation, lower or higher precipitation, and forests, whereas it was negatively associated with lower or higher elevation. The incidence of hospitalization for SFTS was positively associated with longer solar radiation and forests but negatively associated with shorter solar radiation and lower or higher elevation.

**Table 1.** Modified Poisson Regression Analysis: Tsutsugamushi Disease

Parameter	IRR	95% CI	*P* Value
Temperature			
Low	1.29	.85–1.95	.227
Middle	1 [Reference]		
High	1.63	1.05–2.52	.028
Volume of solar radiation			
Low	0.59	.42–.84	.003
Middle	1 [Reference]		
High	0.90	.60–1.35	.607
Elevation			
Low	0.45	.25–.80	.007
Middle	1 [Reference]		
High	1.44	.88–2.36	.146
Precipitation			
Low	0.56	.42–.76	<.001
Middle	1 [Reference]		
High	1.16	.89–1.51	.284
Vegetation			
Others	1 [Reference]		
Farm	4.46	1.59–12.52	.005
Forest	5.35	1.78–16.07	.003
Random effect	Estimate	SE	
Population	0.066	0.019	

Abbreviation: IRR, incidence rate ratio.

**Table 2.** Modified Poisson Regression Analysis: Japanese Spotted Fever

Parameter	IRR	95% CI	*P* Value
Temperature			
Low	0.06	.02–.20	<.001
Middle	1 [Reference]		
High	0.75	.36–1.56	.439
Volume of solar radiation			
Low	0.69	.30–1.60	.388
Middle	1 [Reference]		
High	3.29	1.44–7.54	.005
Elevation			
Low	0.31	.11–.86	.024
Middle	1 [Reference]		
High	0.31	.16–.58	<.001
Precipitation			
Low	3.86	1.54–9.69	.004
Middle	1 [Reference]		
High	2.66	1.17–6.07	.020
Vegetation			
Others	1 [Reference]		
Farm	1.46	.72–2.96	.289
Forest	11.40	4.97–26.13	<.001
Random effect	Estimate	SE	
Population	0.037	0.048	

Abbreviation: IRR, incidence rate ratio.

**Table 3.** Modified Poisson Regression Analysis: Severe Fever With Thrombocytopenia Syndrome

Parameter	IRR	95% CI	*P* Value
Temperature			
Low	0.22	.15–.35	<.001
Middle	1 [Reference]		
High	1.40	.96–2.06	.084
Volume of solar radiation			
Low	0.40	.23–.71	.002
Middle	1 [Reference]		
High	1.81	1.20–2.72	.004
Elevation			
Low	0.28	.15–.51	<.001
Middle	1 [Reference]		
High	0.57	.35–.93	.024
Precipitation			
Low	1.01	.60–1.71	.957
Middle	1 [Reference]		
High	1.39	.94–2.07	.100
Vegetation			
Others	1 [Reference]		
Farm	2.37	.63–12.17	.178
Forest	13.65	4.03–46.20	<.001
Random effect	Estimate	SE	
Population	0.024	0.030	

Abbreviation: IRR, incidence rate ratio.

## DISCUSSION

Environmental factors of patient residences, such as temperature and humidity, are important determinants of the development, survival, and activity of ticks, especially in the Lyme disease vector *Ixodes*, and have been reported [19–26]. In our study, based on enrollment in the DPC, we analyzed the association of patients with Japanese spotted fever, tsutsugamushi disease, and SFTS and their residential areas, by factors such as ambient temperature, sunshine hours, elevation, precipitation, and green space. The results showed that the temperature, sunshine hours, elevation, precipitation, and green space areas of patient residences were associated with an increased risk of infectious diseases.

Our analysis revealed that a shorter duration of sunlight, lower elevation, and less rainfall were associated with a lower risk of contracting tsutsugamushi disease, while agricultural and forested areas were associated with a higher risk of contracting the disease. From 2006 to 2018, Luo et al reported a positive correlation between the incidence of tsutsugamushi disease in Xiamen City, China, and several weather factors, including average temperature, solar radiation, and precipitation [27]. Similarly, Yang et al noted increases in the monthly number of cases of tsutsugamushi disease: 4% to 15% for each additional 1 °C increase in average monthly temperature over the past 3 months, 6% to 12% for an additional 1% increase in monthly relative humidity over the past 2 months, and 0% to 7% an additional 1-mm increase in monthly precipitation over the past 3 months [28]. Several other studies have found that temperature, humidity, and rainfall are associated with the risk of tsutsugamushi disease transmission [29]. To our knowledge, this is the first study to show that environmental factors are associated with the risk of tsutsugamushi disease in Japan.

For Japanese spotted fever, no studies have analyzed the association between the risk of infection and environmental factors. In Japan, erythrocytic fever cases are concentrated in the western part of the country, which is thought to be due to the influence of temperature and climate on tick survival in these regions.

SFTS, an emerging tick-borne infectious disease caused by the SFTS virus, was first reported in China in 2009 [30]. Cases have been documented in Japan since 2013, primarily in western Japan [6]. Our study showed that the risk of SFTS was higher in areas with long daylight hours and in forested areas. Deng et al [31] reported that the incidence of SFTS increased when the daylight duration was >11 hours per day and decreased when the temperature was high (>28 °C), which is consistent with our results regarding daylight hours.

The present study has some limitations. First, the number of cases of tick-borne infections may be underestimated for the following reasons. (1) The database used in this study does not include an exhaustive data set of patients from all acute care beds in Japan. (2) The validity of diagnosis based on *ICD-10* codes for tick-borne infections in this database has not been verified. (3) Not all patients with tick-borne infections were hospitalized; some were treated on an outpatient basis. Second, although this study used data on patients’ residential areas, not all patients would have been bitten by ticks at their residence. Some patients could have been bitten away from their residential area, such as the patients’ house, which could have led to misclassification. Third, several geospatial and weather data aggregated at each city level by Japanese local government code do not represent exact patient residence information. Some patients may live close to where they become infected, while others usually get infected by going out into mountains, forests, or even islands far from their residential areas. Therefore, there is a risk of misclassifying the geographic characteristics of places of infection. However, when patients visit areas within the same city code or close to the city code where they reside, the association remains. Fourth, coinfections were not investigated in our database. Several articles have reported mixed infections of SFTSV and scrub typhus [32, 33]. In addition, a report from China revealed that SFTSV has been detected by reverse transcription polymerase chain reaction in *Leptotrombidium scutellare* mites [34]. Thus, some ticks may vector SFTSV and scrub typhus, but these coinfections were not analyzed in our research.

## CONCLUSION

Our study revealed the relationship of the risk of Japanese spotted fever, tsutsugamushi disease, and SFTS with the characteristics of environmental factors. These results may help identify areas at high risk of infection transmission, which will be useful in educating the public on infection prevention.

## Supplementary Material

ofae025_Supplementary_Data

## Supplementary Data

Supplementary materials are available at *Open Forum Infectious Diseases* online. Consisting of data provided by the authors to benefit the reader, the posted materials are not copyedited and are the sole responsibility of the authors, so questions or comments should be addressed to the corresponding author.

## Notes

***Author contributions.*** S. K. conceived the study. H. O. curated the data. T. O. and S. T. analyzed the data. H. M., K. F., and H. Y supervised the study. All authors interpreted the results, wrote the first draft of the manuscript, and critically reviewed it. All authors agreed with the final version of the manuscript.

***Data availability.*** Data are not publicly available.

***Ethical approval statement.*** The study was conducted in accordance with the principles of the Declaration of Helsinki and approved by the Institutional Review Board of the University of Tokyo (approval 3501-3; 25 December 2017). The requirement for obtaining informed consent was waived owing to the retrospective nature of the study and the use of anonymized data.

***Financial support.*** This work was supported by grants from the Japan Agency for Medical Research and Development (22fk0108625h0701) and was conducted as part of the All-Osaka U Research in “The Nippon Foundation–Osaka University Project for Infectious Disease Prevention.”
