# Supplementary material for: Analysis of Differences in Characteristics of High-Risk Endemic Areas for Contracting Japanese Spotted Fever, Tsutsugamushi Disease, and Severe Fever With Thrombocytopenia Syndrome
Source: Open Forum Infect Dis. 2024 Jan 16;11(2):ofae025. doi: 10.1093/ofid/ofae025 (PMC10836194; doi:10.1093/ofid/ofae025)
Supplement: ofae025_Supplementary_Data [file ofae025_supplementary_data.zip › Suppliment table_v2.docx]

**Supplement Table 1. ICD-10 codes and diagnosis names**

| **ICD-10 code** | **Diagnosis name** | **Counts** |
| --- | --- | --- |
| A68 | Relapsing fevers | 15 |
| A69 | Other spirochetal infections | 157 |
| A75 | Typhus fever | 1666 |
| A77 | Spotted fever [tick-borne rickettsioses] | 1417 |
| A79 | Other rickettsioses | 576 |
| A84 | Tick-borne viral encephalitis | 5 |
| A93 | Other arthropod-borne viral fevers, not elsewhere classified | 466 |
| B60 | Other protozoal diseases, not elsewhere classified | 98 |
| B83 | Other helminthiases | 382 |

**Supplement Table 2. Definition of each landscape**

| **Variable** | **Definition** |
| --- | --- |
| Forest | Area dominated by trees higher than shrubs with a canopy cover 10 greater than or equal to percent. |
| Farm | Area where agricultural activities are implemented constantly |
| Other | Areas other than above. |

Parameters are defined by International Steering Committee for Global Mapping

**Supplement Table 3. Characteristics of weather and land parameters in each city areas.**

| **Parameter** | **Min– Max** | **Mean** |
| --- | --- | --- |
| Annual average temperature, C |  |  |
| Low temperature | -1.1 – 11.6 | 8.0 |
| Middle temperature | 11.7 – 15.0 | 13.7 |
| High temperature | 15.1 – 23.9 | 16.4 |
| Solar radiation, MJ/m2 |  |  |
| Low volume of solar radiation | 11.0 – 12.8 | 12.3 |
| Middle volume of solar radiation | 12.9 – 13.4 | 13.2 |
| High volume of solar radiation | 13.5 – 15.9 | 13.9 |
| Elevation, m |  |  |
| Low elevation | 1.4 – 9.7 | 4.7 |
| Middle precipitation | 9.8 – 29.7 | 18.8 |
| High elevation | 29.8 – 113.9 | 56.1 |
| Precipitation, mm |  |  |
| Low precipitation | 675 – 1405 | 1217 |
| Middle precipitation | 1406 – 1851 | 1608 |
| High precipitation | 1852 – 3946 | 2331 |
| **Parameter** | **Percentage** | **Counts** |
| Vegetation, n |  |  |
| Vegetation (others) | 65.0 | 1159 |
| Vegetation (farm) | 18.5 | 330 |
| Vegetation (forest) | 16.5 | 293 |

SD; Standard deviation
