# Supplementary material for: Analysis of Differences in Characteristics of High-Risk Endemic Areas for Contracting Japanese Spotted Fever, Tsutsugamushi Disease, and Severe Fever With Thrombocytopenia Syndrome
Source: Open Forum Infect Dis. 2024 Jan 16;11(2):ofae025. doi: 10.1093/ofid/ofae025 (PMC10836194; doi:10.1093/ofid/ofae025)

Supplement Figure1-A. Annual average temperature in each area in Japan over the observational period

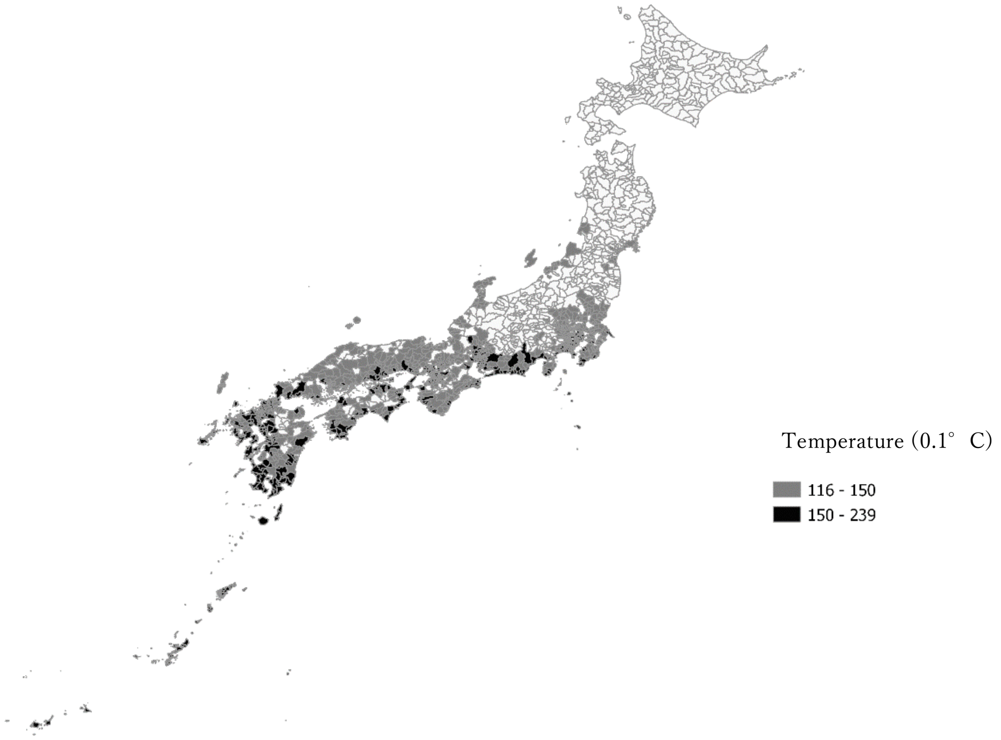

Supplement Figure 1-B. Annual average solar radiation in each area in Japan over the observational period

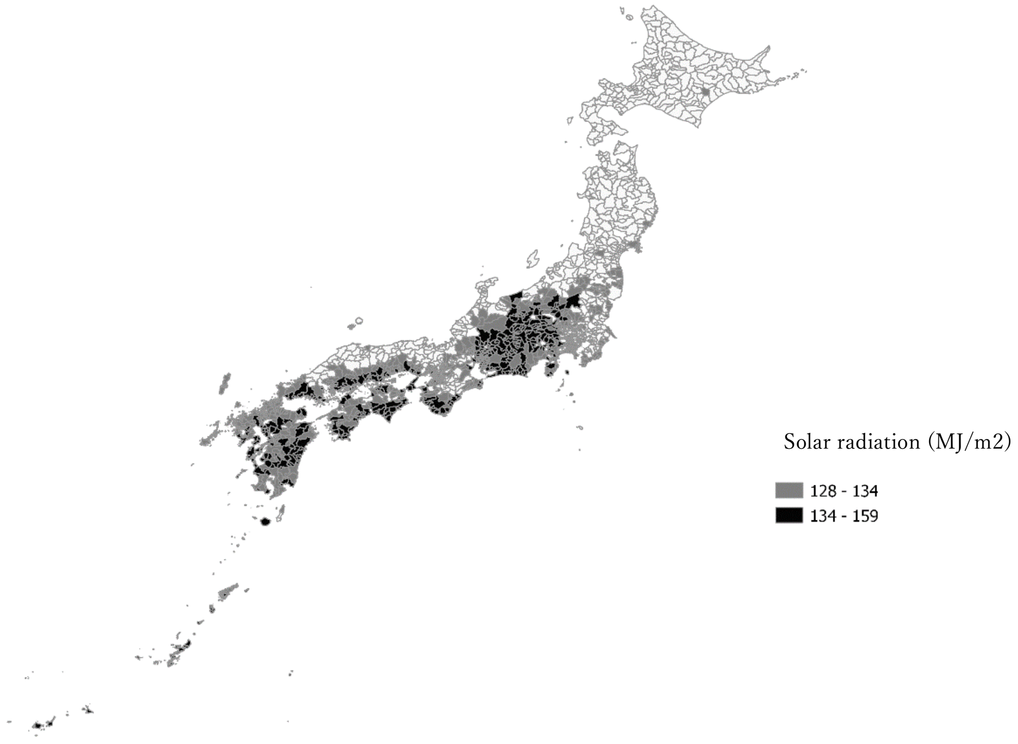

Supplement Figure 1-C. Annual average precipitation in each area in Japan over the observational period

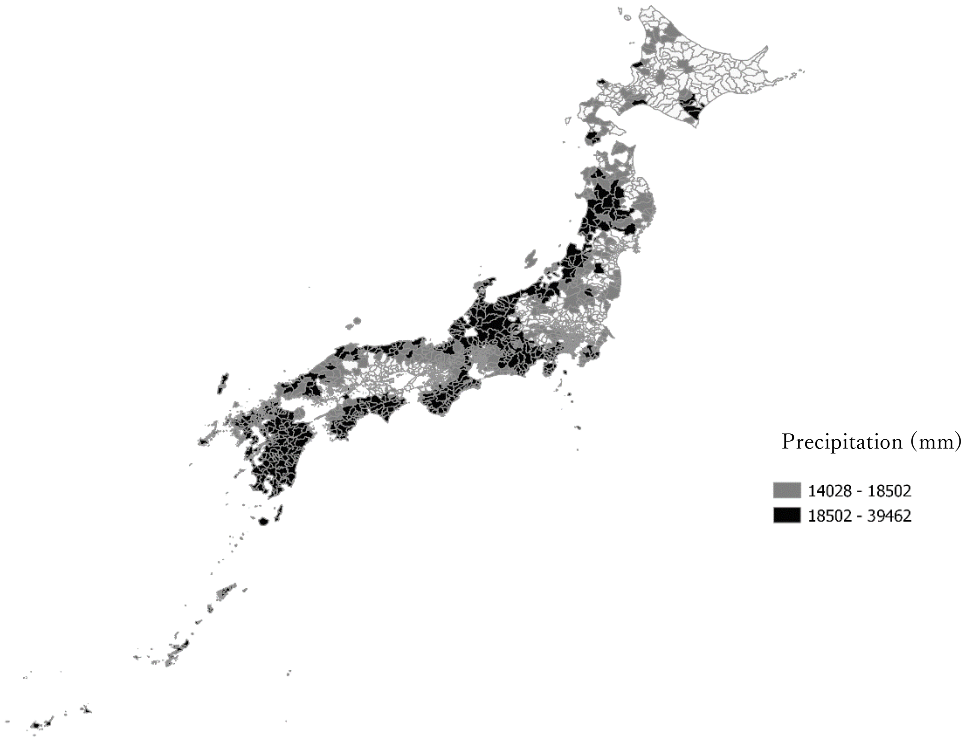

Supplement Figure 2-A. The elevation in each area in Japan over the observational period

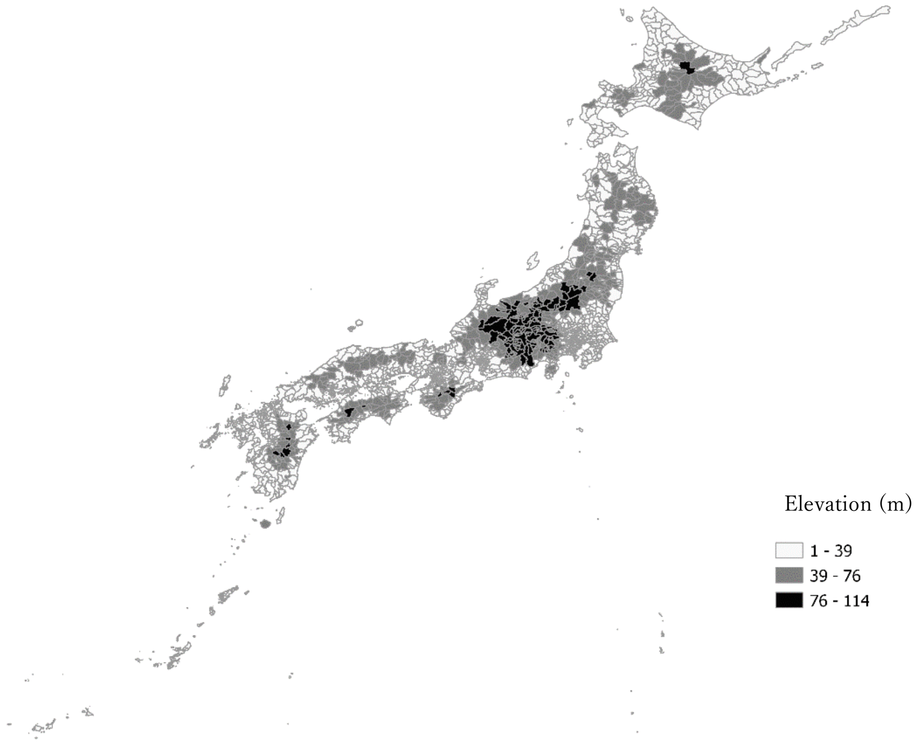

Supplement Figure 2-B. Types of major landscape in each area in Japan over the observational period

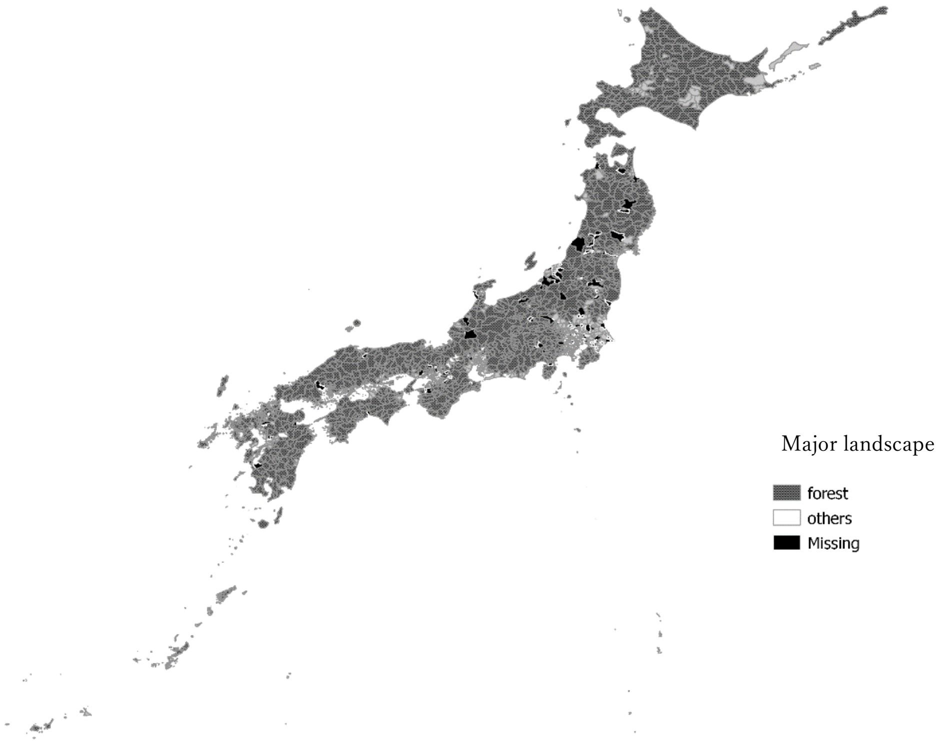

Supplement Figure 2-C. Total population in each area in Japan over the observational period

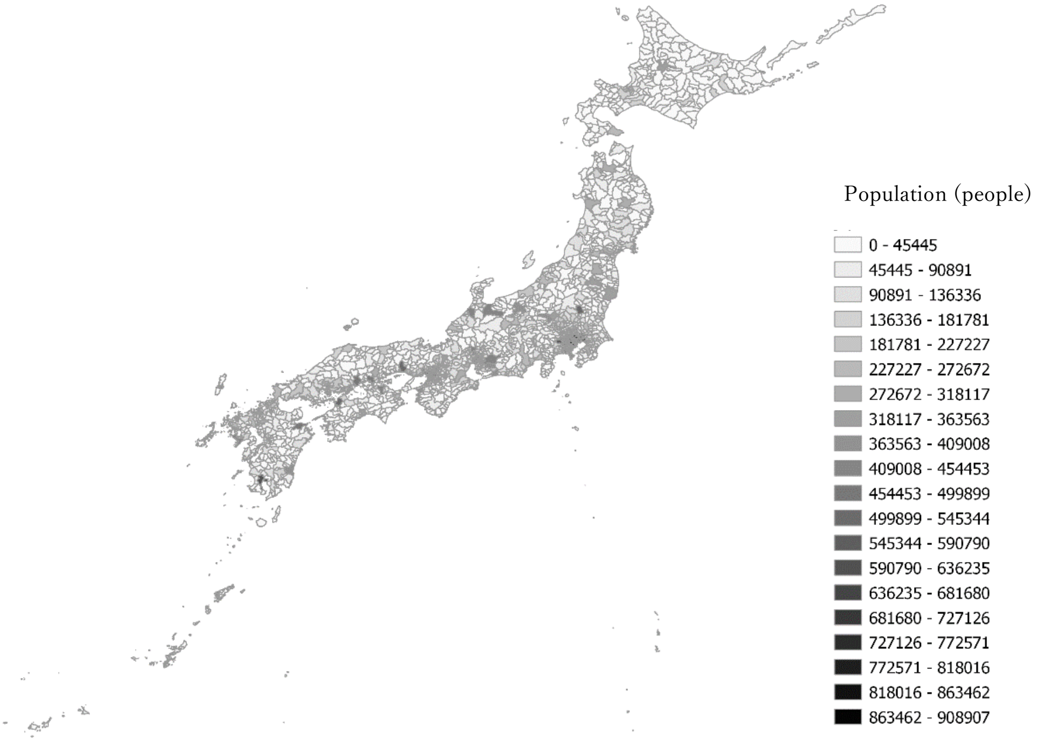

Supplement: ofae025_Supplementary_Data [file ofae025_supplementary_data.zip › Suppliment_v2.pdf]
